# Pure Red Cell Aplasia and Other Haematological Diseases Associated With Thymoma: A Case Series and Systematic Review

**DOI:** 10.3389/fmed.2021.759914

**Published:** 2021-12-13

**Authors:** Chih-Chieh Yen, Wei-Li Huang, Sin-Syue Li, Ya-Ping Chen, Yau-Lin Tseng, Yi-Ting Yen, Chang-Yao Chu, Ya-Ting Hsu, Tsai-Yun Chen

**Affiliations:** ^1^Division of Haematology/Oncology, Department of Internal Medicine, National Cheng Kung University Hospital Douliou Branch, Yunlin, Taiwan; ^2^Institute of Clinical Medicine, School of Medicine, National Cheng Kung University, Tainan, Taiwan; ^3^Division of Thoracic Surgery, Department of Surgery, National Cheng Kung University Hospital, College of Medicine, National Cheng Kung University, Tainan, Taiwan; ^4^Division of Haematology, Department of Internal Medicine, National Cheng Kung University Hospital, College of Medicine, National Cheng Kung University, Tainan, Taiwan; ^5^Department of Pathology, Chi-Mei Medical Center, Tainan, Taiwan

**Keywords:** thymoma, thymectomy, systematic review, pure red cell aplasia, autoimmune disease, Good's syndrome

## Abstract

**Background:** Thymoma-associated haematological diseases (HDs), such as pure red cell aplasia (PRCA) and Good's syndrome, are extremely rare, and due to the paucity of large-scale studies, the characteristics, remission after thymectomy, and long-term evaluation remain undetermined.

**Methods:** We retrospectively assessed patients with thymoma and associated HDs from Jan 2005 to Dec 2020. All patients received thymectomy and/or additional treatments for HDs. A comparison with thymoma-associated myasthenic gravis (MG), and a systematic review from PubMed/MEDLINE and Embase were conducted.

**Results:** In the median follow-up of 56 months, 130 patients were enrolled. Patients with thymoma-associated MG (*n* = 46) and HDs [*n* = 8; PRCA (*n* = 5), PRCA and Good's syndrome (*n* = 2) and autoimmune haemolytic anaemia (*n* = 1)] were evaluated. Patients with MG had a significantly higher remission rate after thymectomy (50 vs. 17%; *p* = 0.0378) as compared to those with other autoimmune diseases. Two of seven patients with PRCA experienced remission with thymectomy alone, and an additional two patients achieved remission with thymectomy plus immunosuppressive therapy (IST). In the systematic review, 60 studies (case reports, *n* = 46; case series including the present study, *n* = 14) were evaluated. Forty-four percent of patients were diagnosed with PRCA after thymoma, and 61% achieved remission with thymectomy plus IST; however, Good's syndrome was unaffected.

**Conclusions:** Our study indicates that patients with thymoma-associated autoimmune diseases other than MG have a lower remission rate than those with MG. Remission of thymoma-associated PRCA can be achieved by thymectomy and IST. This study provides insight into extremely rare but puzzling autoimmune manifestations.

## Introduction

Thymoma is a rare mediastinal tumour that originates from the epithelium of the thymus, with an incidence of 0.13–0.15 per 100,000 person-years (1, 2). Thymoma correlates with various autoimmune diseases, among which myasthenia gravis (MG) is the most prevalent and is found in up to 44% of cases (3, 4). Other autoimmune diseases, especially haematological diseases (HDs), are less frequently reported but may affect survival (5–7). Unlike thymoma-associated MG, surgical thymectomy for other autoimmune diseases remains under debate owing to the largely unknown efficacy and risks of disease progression (8, 9). Overall, there is a knowledge gap regarding the aetiological association between thymoma and autoimmune diseases, notably those other than MG.

Pure red cell aplasia (PRCA) is a rare acquired isolated erythropoietic failure and one of the most common thymoma-associated HDs. The incidence of PRCA is 2–5% in patients with thymoma, and a thymic tumour can be detected in 10–20% of patients with PRCA, suggesting autoimmune interaction (10–12). Thymoma-associated PRCA often accompanies other HDs, including Good's syndrome (thymoma plus hypogammaglobulinaemia), autoimmune haemolytic anaemia (AIHA) and other cytopaenias (11, 13). Unlike MG, remission of PRCA after thymectomy has yet to be determined, and several series have inconsistently reported remission rates of 5–25% (11, 14, 15). Interestingly, B cell aplasia and hypogammaglobulinaemia in Good's syndrome are largely unaffected by immunosuppressive therapy (IST) and thymectomy (13). Differences in characteristics, therapeutic responses, and chronological sequences highlight the complex interplay between thymoma and HDs.

Thymoma-associated PRCA and other HDs are to date only found in case reports or series. The disease trajectory and outcome of patients who have undergone thymectomy are also less reported. Hence, a systematic review may provide insight into rare but potentially devastating manifestations. In the present study, we retrospectively reviewed thymoma-associated HDs in a medical institute. A descriptive analysis was conducted to elucidate the characteristics, chronological sequences, treatments, remissions, and outcomes of these patients. The aim of the study was to reveal the features and long-term evaluation of thymoma-associated PRCA and other HDs. A systematic review incorporating case reports and series in the past 15 years is provided as well.

## Materials and Methods

### Patients

We retrospectively reviewed patients with primary thymic epithelial tumours from Jan 2005 to Dec 2020 at National Cheng Kung University Hospital, a tertiary medical institute in Tainan, Taiwan. A total of 130 patients who had follicular hyperplasia, thymoma, or thymic carcinoma and had received thymectomy (thoracotomic or video-associated thoracoscopic thymectomy) were enrolled. All tumour specimens were uniformly confirmed by a pathologist (CY Chu) specialising in chest malignancies according to the 2015 WHO Classification of Tumours of the Thymus (4th edition) (16). Requirement of additional therapies for thymic malignancies, such as systemic chemotherapy and radiotherapy, did not affect inclusion. Patients with thymic epithelial tumours other than thymoma, such as germ cell tumours, soft tissue sarcomas, secondary lymphomas, and other haematopoietic neoplasms, were excluded. Associated autoimmune diseases, including HDs (PRCA, Good's syndrome, and AIHA), MG, Graves' disease and other inflammatory peripheral neuropathies, were assessed based on electronic medical records. The diagnosis of PRCA was established as follows: the presence of severe normocytic, normochromic anaemia with reticulocytopaenia and the absence or near absence of erythroid precursors (<1% in the total nucleated cells) in otherwise normal bone marrow. Good's syndrome was diagnosed according to primary hypogammaglobulinaemia [serum immunoglobulin G (IgG) <500 mg/L, IgA <700 mg/L, IgM <400 mg/L, and/or IgG subclass deficiency] and AIHA to the presence of autoimmune antibody-mediated haemolysis plus positive direct/indirect anti-globulin test (17–19). Patients with HDs derived from congenital or any secondary causes other than thymoma were excluded.

### Clinical Evaluations

The clinicopathological characteristics of the patients were recorded. We used the Masaoka-Koga staging system for thymic malignancies (20). The selected treatments for PRCA, such as corticosteroids, cyclosporin A (CsA), cyclophosphamide (CYC), intravenous immunoglobulin (IVIg), antithymocyte globulin (ATG), and/or other therapeutic monoclonal antibodies, were noted accordingly. Complete remission (CR) of PRCA was referred to as a persistent haemoglobin (Hb) level ≥11.0 g/dL and partial remission (PR) as Hb between 9.0 and 11.0 g/dL over a period of 3 months. Non-remission (NR) was defined by any condition that did not fit the above criteria. The chronological sequence of PRCA and thymoma was defined by either disease preceding the other with an interval of more than 3 months; concurrence was suggested if two diseases were diagnosed with an interval ≤3 months. Transfusion dependence was defined by at least 2 units of red blood cell transfusions every 28 days over a period of 3 months. Overall survival was defined as the interval between thymoma diagnosis and death of the patient for any cause.

### Search Strategies and Systematic Review of the Literature

**Figure 1:**
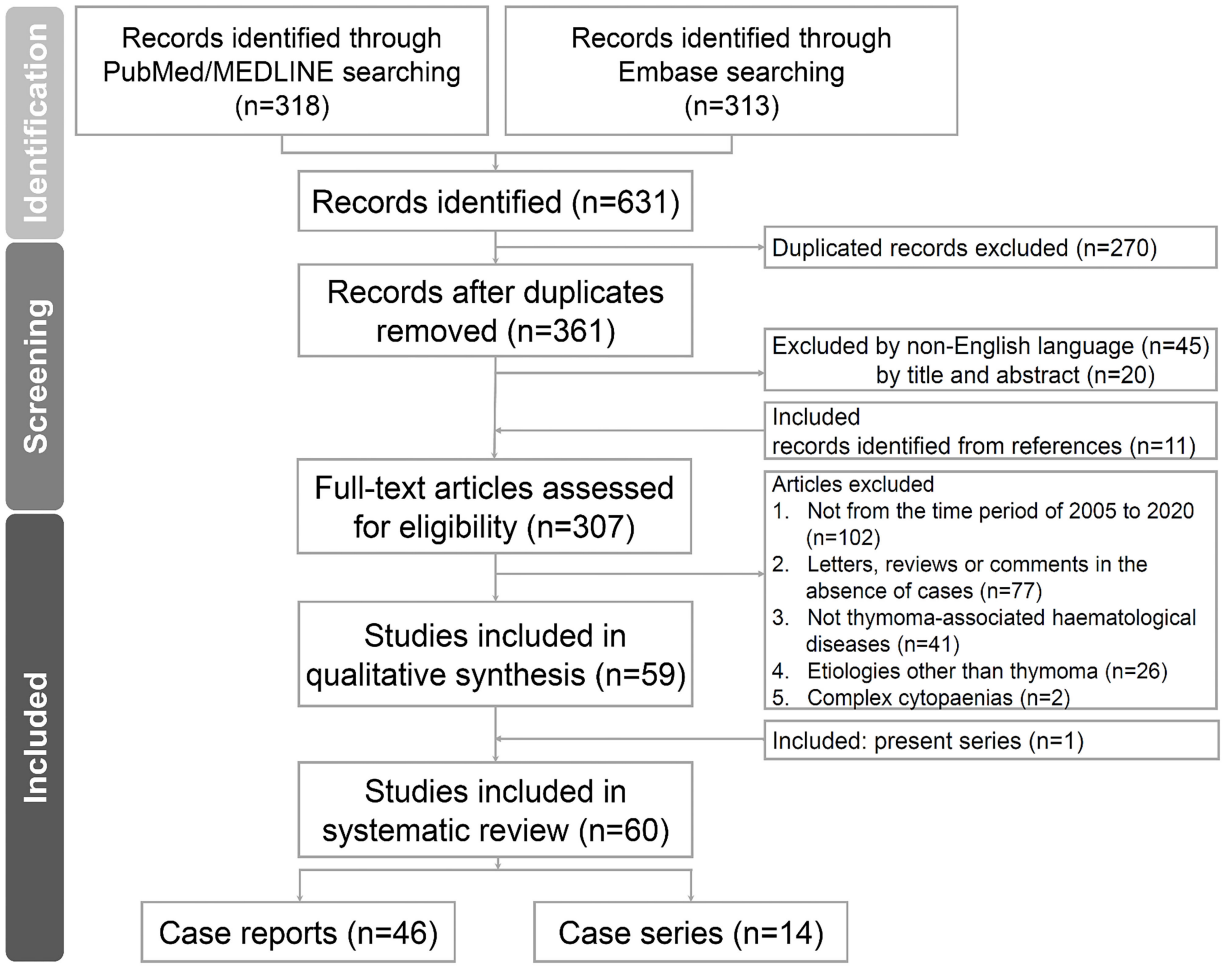
Flow diagram of the study. The flow diagram of the enrolled studies following PRISMA algorithm. PRISMA, preferred reporting items for systematic reviews and meta-analyses.

We conducted a systematic review of the literature on thymoma-associated PRCA. Two authors (CC Yen and YT Hsu) independently performed literature searches in online databases, including PubMed/MEDLINE and Embase, in February 2021. The search keywords included “THYMOMA,” “THYMIC EPITHELIAL TUMOUR,” and “PURE RED CELL APLASIA” by controlled or natural vocabularies, respectively. Other cytopaenias in addition to PRCA, such as Good's syndrome or AIHA, were not excluded from our search results and were therefore assessed collectively. The details of the search queries are shown in Supplementary Data 1. We further limited the preliminary searches to the English language, research articles of case reports, series, observational or prospective studies, and publication year from 2005 to 2020. We followed the Preferred Reporting Items for Systematic Reviews and Meta-Analyses (PRISMA) for qualitative assessment (21). The flow algorithm of the searched articles is depicted in Figure 1. If the two authors failed to reach a consensus, an independent third author (SS Li) was summoned for a final decision. The PRISMA checklist of the current study is provided in Supplementary Data 2.

### Statistical Analysis

We present clinicopathological features in descriptive analyses by percentages or case numbers. Continuous variables were compared using Student's *t*-test; categorical variables were compared using a chi-squared test or Fisher's exact test. If variables did not meet the parametric assumptions, non-parametric methods were applied. Statistical significance was prespecified by a *p* < 0.05. In pooled analysis, we aggregated the available patient data as much as possible and did not impute missing information. Because of publication bias, we did not restrict data derived from published variables in the articles but used all searchable information. We employed GraphPad Prism^®^ 8.0 (GraphPad software, CA, USA) and R^®^ 3.5.1 for data management and computing.

## Results

### Patients With Thymoma: Clinicopathological Characteristics

A total of 130 patients with thymoma who had received thymectomy were included in the study. Their clinicopathological characteristics are shown in Table 1. The median follow-up time was 56 months, and the median age was 56 years, with a female predominance (57%). Nearly half (45%) of the patients had at least one associated autoimmune disease, with MG being the most prevalent (36%), followed by HDs (PRCA, 5%; Good's syndrome, 2%; AIHA, 1%). Cortical (WHO B2, 22%) and mixed (WHO AB, 22%) thymoma were frequently found based on WHO classification. Fourteen percent of the patients had thymic carcinoma, and 6% had follicular hyperplasia. In approximately half of the cases, a thymoma with extracapsular invasion or distant metastasis occurred (Masaoka stage IIA or above, 56%). However, successful en bloc resection of tumours was achieved in 99% of the patients.

**Table 1:** Patient characteristics.

	**Patients with thymoma (*****n*** **= 130)**	
Age at diagnosis, years (IQR)	53	(44–63)
Male, *n* (%)	56	(43)
Median follow-up duration, ms (IQR)	54	(27–86)
With at least one AD, *n* (%)	58	(45)
With ≥2 Ads	4	(3)
Thymoma and ADs^a^		
With MG	47	(36)
With PRCA	7	(5)
With Good's syndrome	2	(2)
Others^b^	5	(4)
WHO classification		
A	17	(13)
B1	18	(14)
B2	28	(22)
B3	13	(10)
AB	28	(22)
C	18	(14)
Follicular hyperplasia	8	(6)
Masaoka staging^c^		
I	54	(44)
II	31	(25)
III	24	(20)
IV	13	(11)
Tumour diameter ≥5 cm, *n* (%)	54	(42)
Angiolymphatic invasion, *n* (%)	8	(6)
En-bloc resection, *n* (%)	128	(99)

a *Calculated as the number of patients with defined AD in the total population*.

b *Graves' disease (n = 2), AIDP (n = 2), and AIHA (n = 1)*.

c *Excluding patients with follicular hyperplasia (n = 122)*.

*IQR, interquartile range; ms, months; AD, autoimmune disease; MG, myasthenia gravis; PRCA, pure red cell aplasia; AIDP, acute inflammatory demyelinating polyneuropathy; AIHA, autoimmune hemolytic anaemia*.

### Patients With MG vs. Other Autoimmune Diseases

In the total cohort, 46 patients had MG, and another 12 had autoimmune diseases other than MG. The detailed characteristics are presented in Table 2. Among patients with autoimmune diseases other than MG, two-thirds had HD (PRCA, *n* = 5; PRCA plus Good's syndrome, *n* = 1; PRCA, Good's syndrome and AIHA, *n* = 1; AIHA, *n* = 1). Overall, sex, en bloc resection rate, concurrence with thymoma, WHO histology classification and Masaoka staging were comparable between the two groups. However, patients with MG were significantly younger than those with other autoimmune diseases (median age, 47 vs. 54 years; *p* = 0.0112 by Mann-Whitney *U*-test), and the remission rate after thymectomy was significantly higher in these patients (50 vs. 17%; *p* = 0.0378 by Fisher's exact test).

**Table 2:** Myasthenia gravis vs. other autoimmune diseases.

	**MG only (*****n*** **= 46)**		**ADs other than MG (*****n*** **= 12)**		***P***
Median age, years (IQR)	47	(35, 53)	54	(49, 65)	0.0112^*^
Male, *n* (%)	15	(33)	5	(42)	0.81
Haematological ADs, *n* (%)	0		8	(67)	-
En-bloc resection, *n* (%)	45	(98)	11	(92)	0.37
Median follow-up durations, ms (IQR)	60	(29, 85)	46	(29, 85)	0.88
Concurrence with thymoma, *n* (%)	18	(39)	4	(33)	0.97
WHO Histology					0.18
A	5	(11)	2	(17)	
B1	5	(11)	0		
B2	14	(30)	2	(17)	
B3	6	(13)	0		
AB	10	(22)	5	(42)	
C	0		1	(8)	
Follicular hyperplasia	6	(13)	2	(17)	
Masaoka^a^					0.78
I	20	(50)	5	(50)	
II	11	(28)	4	(40)	
III	6	(15)	1	(10)	
IV	3	(8)	0		
Remission after thymectomy, *n* (%)	23	(50)	2	(17)	0.0378^*^

a *Excluding patients with follicular hyperplasia (MG alone, n = 40; ADs other than MG, n = 10)*.

*MG, myasthenia gravis; AD, autoimmune disease; IQR, interquartile range; ms, month*.

* *p < 0.05 (statistically significant)*.

### Patients With Thymoma and PRCA: Clinical Course

In the median follow-up of 54 months, seven patients had thymoma-associated PRCA and other HDs. Five of the patients had PRCA, one had PRCA plus Good's syndrome and one had PRCA, Good's syndrome plus AIHA. The detailed clinical course, characteristics and illustrated chronological sequence of the diseases are provided in Table 3 and Supplementary Figure 1. We present the clinicopathological features of a middle-aged female (Case 1) with thymoma-associated PRCA in Supplementary Figure 2. In the chronological sequence of the two diseases, five patients were found to have a concurrent course, followed by one patient who had PRCA preceding thymoma and one who had thymoma preceding PRCA. Initially, two patients achieved remission of PRCA by thymectomy. Among the 7 included patients, four achieved a treatment response above PR (PR, *n* = 2; CR, *n* = 2). Three of the seven patients had achieved a remission by thymectomy and IST with corticosteroids, CsA, CYC, IVIg, or ATG (PR, *n* = 2; CR, *n* = 1); two had NR; one had a sustained CR without IST for a follow-up time of 130 months, and one was not evaluable due to early death. Patients with Good's syndrome failed to respond to these treatments and were supported with IVIg in the follow-up period.

**Table 3:** Patient characteristics of the present series.

	**Age (range), sex**	**ADs**	**WHO histology**	**Masaoka**	**Pre/post thymectomy Hb, g/dL**	**Sequence of thymoma and PRCA^a^**	**Remission after thymectomy**	**Frontline treatment**	**Relapse or progression**	**Additional treatment(s)**	**FUT, ms**	**Outcome**
**1**	50–55 F	PRCA, PGA type 1	B1	IIA	6.5; 6.4	Concurrent	No	CsA, corticosteroids	Yes, by 18 ms	CYC, IVIg^b^, rATG	46	Alive with PR, Tr-D (+) due to 2nd relapse
**2**	50–55 F	PRCA, Good's syndrome	AB	I	4.5; 4.8	Thymoma preceding by 22 ms	No	CsA, corticosteroids	No	IVIg^b^	54	Alive with CR, Tr-D (–)
**3**	50–55 F	PRCA, Good's syndrome, AIHA	AB	I	5.8; 6.2	Concurrent	No	None	Yes	IVIg^b^	1	Not evaluable; death due to sepsis; Tr-D (+)
**4**	55–60 M	PRCA, MG	A	I	5.0; 5.9	Concurrent	No	CsA, corticosteroids	No	None	25	Alive with NR, Tr-D (–)
**5**	60–65 F	PRCA	AB	I	5.7; 10.1	Concurrent	Yes	None	No	None	130	Alive with CR, Tr-D (–)
**6**	50–55 M	PRCA	AB	IIA	4.4; 14.4	PRCA preceding by 4 ms	Yes	None	Yes, by 78 ms	CsA, corticosteroids	138	Alive with PR, Tr-D (+); developed T-LGL leukaemia
**7**	60–65 M	PRCA	B2	IIA	4.3; 6.4	Concurrent	No	None	Yes, by 4 ms	None	22	NR; death due to myeloid malignancy; Tr-D (+)

a *Concurrent: the time interval between diagnosis of thymoma and PRCA equivalent or <3 months*.

b *IVIg administration as a prophylactic treatment for hypogammaglobulinaemia*.

*PRCA, pure red cell aplasia; FUT, follow-up time; AIHA, autoimmune haemolytic anaemia; ADs, autoimmune diseases; Hb, haemoglobin; ms, months; CR, complete remission; PR, partial remission; NR, none remission; Tr-D, transfusion dependence; PGA, autoimmune polyglandular syndrome; CsA, cyclosporin A; CYC, cyclophosphamide; IVIg, intravenous immunoglobin; rATG, rabbit anti-human thymocyte globulin; T-LGL, T cell-large granular lymphocyte*.

Two patients in our series developed secondary HDs. One patient had T-large granular lymphocytic leukaemia at 51 months after the diagnosis of PRCA, and circulating large granular lymphocytes persisted throughout the follow-up period. Another patient developed myeloid malignancy with leukaemic transformation at 6 months after PRCA diagnosis. In our series, one patient died of severe sepsis due to Good's syndrome and another of myeloid leukaemia disease progression.

### Systematic Review of the Literature

We identified 307 independent articles about thymoma-associated PRCA or PRCA plus additional HDs in online databases. After screening and assessment for eligibility, 60 articles were included in the systematic review (case series including the present study, *n* = 14; case reports, *n* = 46). The flow diagram according to the PRISMA guidelines is illustrated in Figure 1, and the studies are shown in Supplementary Table 1. Among these studies, we selected five case series for comparisons (Thompson 2006, Hirokawa 2008/2015, Rivoisy 2016, Moriyama 2018, and present study; Table 4) (11, 15, 22–24). Overall, the patient characteristics and selected treatments were similar. In addition to thymectomy, corticosteroids, CsA, ATG, CYC, mycophenolate mofetil, rituximab, alemtuzumab, and danazol were reported as PRCA treatment. The outcomes were also similar, and fatal cases related to infections were found in all 5 series.

**Table 4:** Comparison of the present study with the literature.

**References**	**Cases**	**mAge/** **Sex**	**HDs**	**mHb, g/dL**	**WHO Histology**	**Masaoka**	**Treatment**	**Remission of the HDs**	**Chronological sequence**	**Outcome**	**Note**
Thompson and Steensma (15)	13	65; M:F = 7:6	PRCA (*n* = 13)	6.1	AB (*n* = 4), B1 (*n* = 2), A (*n* = 2), C (*n* = 1); others (*n* = 4)^a^	NA	Thymectomy (*n* = 12), corticosteroids (*n* = 1), ATG/corticosteroids (*n* = 1), ATG/CsA (*n* = 2)	Yes (≥PR, *n* = 4), no (*n* = 8)	Thymoma preceded (*n* = 4), concurrent (*n* = 9)	Death due to infection (*n* = 2), progressive thymoma (*n* = 1), bleeding (*n* = 1), heart failure (*n* = 1), NA (*n* = 2)	CsA-related TTP (*n* = 2)
Hirokawa et al. (11, 22)	41	65; M (44%)	PRCA (*n =* 40), PRCA and AIHA (*n =* 1)	5.8	AB (*n =* 9), B2 (*n =* 4), B1 (*n =* 3), A (*n =* 1); others (*n =* 24)^b^	NA	Thymectomy (*n =* 36), CsA (*n =* 20), corticosteroids (*n =* 13), and CYC (*n =* 1)	Yes (≥PR, *n =* 27), no (*n =* 8), NA (*n =* 6)	Thymoma preceded (*n =* 16), PRCA preceded (*n =* 11), unknown (*n =* 14)	Median OS = 142 months Death due to infections (*n =* 5), thymoma (*n =* 1), heart failure (*n =* 1)	IST-related pneumonia (*n =* 4), CMV (*n =* 1)
Rivoisy et al. (23)	11/36^c^	57; M:F = 4:7	PRCA (*n =* 11)	7.0	A (3%), B1-3 (47%), AB (25%), C (14%), NA (11%)^d^	I-II (50%)^d^	Thymectomy (*n =* 11), corticosteroids (*n =* 13), ATG (*n =* 1), CsA (*n =* 9), CYC (*n =* 1), MMF (*n =* 2), rituximab (*n =* 1), alemtuzumab (*n =* 1), danazol (*n =* 2)	Yes (≥PR, *n =* 9), no (*n =* 2)	Thymoma preceded (*n =* 7), concurrent (*n =* 3), PRCA preceded (*n =* 1)	Death due to cytopenia (*n =* 1) and infection (*n =* 1)	IST-related VZV, CMV, and pneumocystis infections
Moriyama et al. (24)	8	56; M:F = 5:3	PRCA (*n =* 7), PRCA and ITP (*n =* 1)	NA	B2 (*n =* 5), B3 (*n =* 2), A (*n =* 1)	II (*n =* 1), III (*n =* 3), IVA (*n =* 2), IVB (*n =* 2)	Thymectomy (*n =* 8), CsA (*n =* 6), corticosteroids (*n =* 2)	Yes (≥PR, *n =* 3), no (*n =* 5)	Thymoma preceded (*n =* 5), concurrent (*n =* 2), PRCA preceded (*n =* 1)	Death due to infection (*n =* 4), heart failure (*n =* 1)	CsA-related pneumonia (*n =* 4) and RI (*n =* 1)
Present study	7	55; M:F = 3:4	PRCA (*n =* 5), PRCA and Good's syndrome (*n =* 1); PRCA, Good's syndrome and AIHA (*n =* 1)	5.0	AB (*n =* 4), B1 (*n =* 1), B2 (*n =* 1), A (*n =* 1)	I (*n =* 4), IIA (*n =* 3)	Thymectomy (*n =* 7), CsA (*n =* 3), corticosteroids (*n =* 4), IVIg (*n =* 2), CYC (*n =* 1), ATG (*n =* 1)	Yes (≥PR, *n =* 4), no (*n =* 3)	Concurrent (*n =* 5), thymoma preceded (*n =* 1), PRCA preceded (*n =* 1)	Death due to myeloid malignancy (*n =* 1), infection (*n =* 1)	No remission of Good's syndrome

a *Thymolipoma (n = 4)*.

b *Follicular hyperplasia (n = 1) and unspecified (n = 23)*.

c *Denotes the cases of PRCA/total cases in the study*.

d *Percentages presented by the total cases of thymoma in the study*.

*mAge, median age; HD, haematological disease; mHb, median haemoglobin; PRCA, pure red cell aplasia; NA, not available; ATG, antithymocyte globulin; CsA, cyclosporine A; TTP, thrombotic thrombocytopenic purpura; AIHA, autoimmune haemolytic anaemia; CYC, cyclophosphamide; MMF, mycophenolate mofetil; IST, immunosuppressive therapy; OS, overall survival; VZV, varicella zoster virus; CMV, cytomegalovirus; ITP, immune thrombocytopenic purpura; RI, renal insufficiency; MDS, myelodysplastic syndrome; IVIg, intravenous immunoglobulin*.

After pooling the information from all enrolled studies, a total of 156 patients with thymoma-associated PRCA were evaluated, and the results are shown in Table 5. The median age was 58, without a sex predilection. Twenty-two percent of the patients were reported to have a concomitant HD in addition to PRCA. Mixed (WHO AB, 26%) and unspecified thymoma were the most prevalent tumours according to WHO classification. In the chronological sequence of thymoma and PRCA, thymoma preceded PRCA in 44% of the patients; 30% had a concurrent course. Regarding the treatment outcome of PRCA, 61% of patients achieved remission after thymectomy and other miscellaneous treatments. Nevertheless, we did not identify any patient with Good's syndrome reported to have remission as defined by reversal of hypogammaglobulinaemia or B cell deficiency.

**Table 5:** Pooled analysis of present series and the literature.

	**Patients with thymoma and PRCA (*n* = 156)**	
Age at diagnosis, years (IQR)	58	(47-65)
Sex, *n* (%)		
Male	75	(48)
Female	81	(52)
Thymoma plus, *n* (%)		
PRCA alone	121	(78)
PRCA and Good's syndrome	22	(14)
PRCA and AAMT	9	(6)
PRCA and AIHA	2	(1)
PRCA and ITP	2	(1)
WHO classification		
A	10	(6)
B1	18	(12)
B2	19	(12)
B3	10	(6)
AB	41	(26)
C	9	(6)
Others^a^	5	(3)
Unspecified	44	(28)
Chronological sequence, *n* (%)		
Thymoma preceded	68	(44)
Concurrent	47	(30)
PRCA preceded	24	(15)
Unknown	17	(11)
Remission of PRCA after thymectomy and other treatments, *n* (%)		
	95	(61)
Remission of Good's syndrome, *n* (%)^b^		
	0	

a *Thymolipoma (n = 4) and follicular hyperplasia (n = 1)*.

b *Defined by resolution of hypogammaglobulinemia or B cell deficiency. A total of 14 cases was reported of the response*.

*IQR, interquartile range; PRCA, pure red cell aplasia; ITP, immune thrombocytopenia purpura; AIHA, autoimmune hemolytic anaemia; AAMT, acquired amegakaryocytic thrombocytopenia*.

## Discussion

To the best of our knowledge, the current study represents one of the few published reports about thymoma-associated PRCA in the literature and the first series in Taiwan. We also incorporated a systematic review of the recent decade to define this rare but puzzling autoimmune manifestation. The incidence of thymoma-related PRCA (5%) and Good's syndrome (2%) in our series were consistent with published studies (4, 13, 23, 25). The complexity and scope of autoimmune manifestations were also similar, whereby MG was most common, accounting for nearly one-third of cases, followed by scattered cases of haematological, rheumatological and neurological disorders (4). Remission of autoimmune disease after thymectomy also varied largely between MG and those other than MG in our study. Together, these results indicate that the complex and aberrant immune dysregulation in thymoma may present with distinctive autoimmune manifestations.

Miscellaneous cytopaenias related to thymoma can be observed but are not limited to a specific haematopoietic lineage (23). In our pooled analysis, Good's syndrome, amegakaryocytic thrombocytopaenia, and AIHA in addition to PRCA accounted for 22% of cases. Therapeutic responses varied in different cytopaenias and relapsed or subsequent involvement of additional lineages was found. Conversely, thymectomy alone did not confer high and durable remission of PRCA. Although early reports indicate a remission rate of 25–30% in patients who undergo thymectomy, recent evidence suggests a contradictory result that remission is rarely durable with thymectomy alone (5, 15, 24, 26). For example, Hirokawa et al. reported that although first IST was effective in 74% of thymoma-associated PRCA, only 2 of 35 patients who received IST remained in sustained remission after discontinuation (11). In our series, only two patients responded to thymectomy, and three maintained remission under IST. In addition, we failed to observe patients with Good's syndrome to recover after thymectomy and other treatments. The United Kingdom-Primary Immune Deficiency registry indicated that patients with Good's syndrome fail to respond to thymectomy or IST and require prolonged IVIg prophylaxis, with 95% having complications of infection that lead to a mortality rate of 9% (13). Consistent with previous reports, our results reflect that long-term evaluation of the disease trajectory, such as cytopaenia or Good's syndrome, might be required and that infectious complications should be carefully accounted for in treating these patients.

Patients with MG constitute a distinctive disease population compared with other autoimmune diseases. We found a significantly higher remission rate after thymectomy with MG. Several hypotheses have been proposed to explain the link between thymoma and paraneoplastic autoimmunity, including immune escape, aberrant autoimmune selection, and genetic predisposition (27–29). In general, the discrepant remission rate in response to thymectomy may be explained by the central role of the thymus in MG but not in other HDs. In contrast, more complex cellular and humoural immune dysregulation is observed in HDs such as PRCA and Good's syndrome (30, 31). Thymectomy does not sufficiently ameliorate autoimmune interactions and requires additional immunomodulatory therapies to achieve durable remission.

In the chronological sequence of thymoma and PRCA, our series revealed a concurrent course in five of seven patients and PRCA after diagnosis of thymoma in 1 patient. In contrast, Hirokawa et al. reported that 60% of patients developed PRCA in a median interval of 80 months after the diagnosis of thymoma (11, 22). Our pooled analysis indicated that 44% of patients developed PRCA after thymoma, with 30% having a concurrent course. The discrepancy of the present series with the literature might have resulted from routine image evaluation for detecting asymptomatic thymomas and limited case numbers. Regardless, the results highlight the need for monitoring the development of subsequent HDs, even in patients who had received thymectomy long ago.

In this series, histology subtypes of thymoma varied and were associated with specific autoimmune manifestations. We observed that six of seven patients with thymoma-associated PRCA had cortical (WHO B1 and B2) or mixed (WHO AB) thymoma, and these tumours were abundantly infiltrated by immature terminal deoxynucleotidyl transferase-positive T cells. Similar results were found in pooled analysis, revealing that half of patients with thymoma-associated PRCA had lymphocyte-rich thymoma (WHO B1, B2, or AB). Although early reports have indicated that PRCA is found predominantly with the spindle cell type (WHO A), recent observations suggest that lymphocyte-rich tumours are prevalent (15, 32, 33). Zaman et al. reported a series of 78 patients with Good's syndrome, among whom 10% had concomitant PRCA, and reported that lymphocyte-rich thymoma constituted the majority of patients (72%), consistent with our results (13). Hoffacker et al. proposed that thymoma patients have higher circulating CD45RA^+^ CD8^+^ T cells with lymphocyte-rich thymoma than healthy controls and that autoimmune T cells respond to thymectomy (34). Despite a putative mechanistic correlation, more information is required to delineate the causal relationship between the development of HDs and autoimmune T cells in lymphocyte-rich thymoma.

The merit of the present study is the incorporation of published reports of extremely rare diseases with relatively adequate case numbers. We also describe detailed clinicopathological characteristics and therapeutic outcomes for a considerably long follow-up time. However, some limitations exist. First, this was a retrospective study, which cannot address causal relation disputes prospectively. Second, publication bias with regard to reporting of investigator-preferred and responsive patients was present among the included articles, with a limitation of case reports instead of large observational or collaborative studies. Our future work will focus on nationwide data to elucidate the interaction of thymectomy and associated HDs and predictors of IST efficacy.

In conclusion, we report a case series of thymoma-associated HDs such as PRCA and Good's syndrome in conjunction with a systematic review. Patients with autoimmune diseases other than MG have a lower remission rate after thymectomy than those with MG. Remission of thymoma-associated PRCA can be achieved by thymectomy and IST. Nonetheless, Good's syndrome is unaffected and associated with the risk of infections. Together, these results provide insight into extremely rare but puzzling autoimmune diseases.

## Data Availability Statement

The data that support the findings of this study are available from the Cancer Registry of the Electronic Medical Records in National Cheng Kung University Hospital, but restrictions apply to their availability. The data for the current study were used under specific permission and are not publicly available elsewhere. The data are, however, available from the authors upon reasonable request and with the permission from the corresponding author Prof. Tsai-Yun Chen. Division of Haematology, Department of Internal Medicine, National Cheng Kung University Hospital, College of Medicine, National Cheng Kung University, Tainan, Taiwan. teresa@mail.ncku.edu.tw, No. 168, Sheng-Li Road, Tainan 70403, Taiwan.

## Ethics Statement

The studies involving human participants were reviewed and approved by B-ER-110-170 National Cheng Kung University Hospital. Written informed consent for participation was not required for this study in accordance with the national legislation and the institutional requirements.

## Author Contributions

C-CY and W-LH contributed for writing the manuscript and data management. S-SL and Y-PC assist in the manuscript writing and systematic review of the literature. Y-LT, Y-TY, and C-YC provide critical patient information. Y-TH and T-YC initiate the study design and investigations. All authors contributed to the article and approved the submitted version.

## Conflict of Interest

The authors declare that the research was conducted in the absence of any commercial or financial relationships that could be construed as a potential conflict of interest.

## Publisher's Note

All claims expressed in this article are solely those of the authors and do not necessarily represent those of their affiliated organizations, or those of the publisher, the editors and the reviewers. Any product that may be evaluated in this article, or claim that may be made by its manufacturer, is not guaranteed or endorsed by the publisher.

## References

[1] Falkson CB Bezjak A Darling G Gregg R Malthaner R Maziak DE. The management of thymoma: a systematic review and practice guideline. J Thorac Oncol. (2009) 4:911–9. 10.1097/JTO.0b013e3181a4b8e0 19557895

[2] Engels EA. Epidemiology of thymoma and associated malignancies. J Thorac Oncol. (2010) 5(10 Suppl 4):S260–5. 10.1097/JTO.0b013e3181f1f62d 20859116 PMC2951303

[3] Fujii Y. Thymus, thymoma and myasthenia gravis. Surg Today. (2013) 43:461–6. 10.1007/s00595-012-0318-2 22948665

[4] Bernard C Frih H Pasquet F Kerever S Jamilloux Y Tronc F. Thymoma associated with autoimmune diseases: 85 cases and literature review. Autoimmun Rev. (2016) 15:82–92. 10.1016/j.autrev.2015.09.005 26408958

[5] Masaoka A Hashimoto T Shibata K Yamakawa Y Nakamae K Iizuka M. Thymomas associated with pure red cell aplasia. Histologic and follow-up studies. Cancer. (1989) 64:1872–8. 10.1002/1097-0142(19891101)64:9<1872::AID-CNCR2820640920>3.0.CO;2-0 2507126

[6] Lee WS Heo DS Bang YJ Lee KS Ahn JS Jung CW. Prognostic factors of patients with thymoma. Korean J Intern Med. (1996) 11:40–9. 10.3904/kjim.1996.11.1.40 8882475 PMC4532000

[7] Sonobe M Nakagawa M Ichinose M Ikegami N Nagasawa M Shindo T. Thymoma. Japanese J Thoracic Cardiovasc Surg. (2001) 49:35–41. 10.1007/BF02913121 11233240

[8] Zhang Z Cui Y Jia R Xue L Liang H. Myasthenia gravis in patients with thymoma affects survival rate following extended thymectomy. Oncol Lett. (2016) 11:4177–82. 10.3892/ol.2016.4528 27313762 PMC4888221

[9] D'Andrea V Malinovsky L Ambrogi V Artico M Capuano LG Buccolini F. Thymectomy as treatment of autoimmune diseases other than myasthenia gravis. Thymus. (1993) 21:1–10. 8480342

[10] Sherer Y Bardayan Y Shoenfeld Y. Thymoma, thymic hyperplasia, thymectomy and autoimmune diseases (Review). Int J Oncol. (1997) 10:939–43. 10.3892/ijo.10.5.939 21533467

[11] Hirokawa M Sawada K Fujishima N Nakao S Urabe A Dan K. Long-term response and outcome following immunosuppressive therapy in thymoma-associated pure red cell aplasia: a nationwide cohort study in Japan by the PRCA collaborative study group. Haematologica. (2008) 93:27–33. 10.3324/haematol.11655 18166782

[12] Charles RJ Sabo KM Kidd PG Abkowitz JL. The pathophysiology of pure red cell aplasia: implications for therapy. Blood. (1996) 87:4831–8. 10.1182/blood.V87.11.4831.bloodjournal87114831 8639856

[13] Zaman M Huissoon A Buckland M Patel S Alachkar H Edgar JD. Clinical and laboratory features of seventy-eight UK patients with Good's syndrome (thymoma and hypogammaglobulinaemia). Clin Exp Immunol. (2019) 195:132–8. 10.1111/cei.13216 30216434 PMC6300645

[14] Zeok JV Todd EP Dillon M DeSimone P Utley JR. The role of thymectomy in red cell aplasia. Ann Thorac Surg. (1979) 28:257–60. 10.1016/S0003-4975(10)63116-5 485627

[15] Thompson CA Steensma DP. Pure red cell aplasia associated with thymoma: clinical insights from a 50-year single-institution experience. Br J Haematol. (2006) 135:405–7. 10.1111/j.1365-2141.2006.06295.x 17032177

[16] Marx A Chan JKC Coindre J-M Detterbeck F Girard N Harris NL. The 2015 World Health Organization classification of tumors of the thymus: continuity and changes. J Thorac Oncol. (2015) 10:1383–95. 10.1097/JTO.0000000000000654 26295375 PMC4581965

[17] Means RT Jr. Pure red cell aplasia. Hematology Am Soc Hematol Educ Program. (2016) 2016:51–6. 10.1182/asheducation-2016.1.51 27913462 PMC6142432

[18] Malphettes M Gérard L Galicier L Boutboul D Asli B Szalat R. Good syndrome: an adult-onset immunodeficiency remarkable for its high incidence of invasive infections and autoimmune complications. Clin Infect Dis. (2015) 61:e13–e9. 10.1093/cid/civ269 25828999

[19] Jäger U Barcellini W Broome CM Gertz MA Hill A Hill QA. Diagnosis and treatment of autoimmune hemolytic anemia in adults: Recommendations from the First International Consensus Meeting. Blood Rev. (2020) 41:100648. 10.1016/j.blre.2019.100648 31839434

[20] Detterbeck FC Nicholson AG Kondo K Van Schil P Moran C. The masaoka-koga stage classification for thymic malignancies: clarification and definition of terms. J Thoracic Oncol. (2011) 6:S1710–S6. 10.1097/JTO.0b013e31821e8cff 21847052

[21] Page MJ McKenzie JE Bossuyt PM Boutron I Hoffmann TC Mulrow CD. The PRISMA 2020 statement: an updated guideline for reporting systematic reviews. BMJ. (2021) 372:n71. 10.1136/bmj.n71 33782057 PMC8005924

[22] Hirokawa M Sawada K Fujishima N Teramura M Bessho M Dan K. Long-term outcome of patients with acquired chronic pure red cell aplasia (PRCA) following immunosuppressive therapy: a final report of the nationwide cohort study in 2004/2006 by the Japan PRCA collaborative study group. Br J Haematol. (2015) 169:879–86. 10.1111/bjh.13376 25807974

[23] Rivoisy C Besse B Girard N Lioger B Viallard JF Lega JC. Thymic epithelial tumor-associated cytopenia: a 10-year observational study in France. J Thorac Oncol. (2016) 11:391–9. 10.1016/j.jtho.2015.11.012 26768832

[24] Moriyama S Yano M Haneda H Okuda K Kawano O Sakane T. Pure red cell aplasia associated with thymoma: a report of a single-center experience. J Thorac Dis. (2018) 10:5066–72. 10.21037/jtd.2018.07.14 30233881 PMC6129890

[25] Verley JM Hollmann KH. Thymoma. A comparative study of clinical stages, histologic features, and survival in 200 cases. Cancer. (1985) 55:1074–86. 10.1002/1097-0142(19850301)55:5<1074::AID-CNCR2820550524>3.0.CO;2-T 3967192

[26] Wang W Chen LY Zhao W Ren Y Wang L Li X. Coexistence of pure red cell aplasia and autoimmune haemolytic anaemia associated with thymoma. Acta Haematol. (2020) 143:491–5. 10.1159/000503376 31962320

[27] Shelly S Agmon-Levin N Altman A Shoenfeld Y. Thymoma and autoimmunity. Cell Mol Immunol. (2011) 8:199–202. 10.1038/cmi.2010.74 21317916 PMC4012878

[28] Kyewski BA. Thymic dendritic cells present blood-borne antigens to medullary thymocytes *in vivo*: a possible role in the generation of the T-cell repertoire. Haematol Blood Transfus. (1985) 29:486–91. 10.1007/978-3-642-70385-0_99 3875535

[29] Okumura M Fujii Y Shiono H Inoue M Minami M Utsumi T. Immunological function of thymoma and pathogenesis of paraneoplastic myasthenia gravis. Gen Thorac Cardiovasc Surg. (2008) 56:143–50. 10.1007/s11748-007-0185-8 18401674

[30] Murase T. Bilineage hematopoietic inhibitor and T lymphocyte dysfunction in a patient with pure red cell aplasia, myasthenia gravis and thymoma. Exp Hematol. (1993) 21:451–5. 8440342

[31] al-Mondhiry H Zanjani ED Spivack M Zalusky R Gordon AS. Pure red cell aplasia and thymoma: loss of serum inhibitor of erythropoiesis following thymectomy. Blood. (1971) 38:576–82. 10.1182/blood.V38.5.576.576 4999340

[32] Tseng-tong K Lee-Yung S. Histologic types of thymoma associated with pure red cell aplasia: A study of five cases including a composite tumor of organoid thymoma associated with an unusual lipofibroadenoma. Int J Surg Pathol. (2001) 9:29. 10.1177/106689690100900106 11469342

[33] Yukio Shimosato KM Matsuno Y. Tumors of the Mediastinum (Atlas of Tumor Pathology), Third Series. (1997). p. 110.

[34] Hoffacker V Schultz A Tiesinga JJ Gold R Schalke B Nix W. Thymomas alter the T-cell subset composition in the blood: a potential mechanism for thymoma-associated autoimmune disease. Blood. (2000) 96:3872–9. 10.1182/blood.V96.12.3872.h8003872_3872_3879 11090072

