# Supplementary material for: Pure Red Cell Aplasia and Other Haematological Diseases Associated With Thymoma: A Case Series and Systematic Review
Source: Front Med (Lausanne). 2021 Dec 13;8:759914. doi: 10.3389/fmed.2021.759914 (PMC8710742; doi:10.3389/fmed.2021.759914)
Supplement: Supplementary file 1 [file Data_Sheet_1.pdf]

Supplementary Materials

Data 1 2

Data 2 4

Figure 1 8

Figure 2 9

Table 1 10

Supplementary Data 1: search queries and results

1. PubMed®/MEDLINE

https://pubmed.ncbi.nlm.nih.gov/

Search time: 13/Feb/2021

Search number	Query	Note	Results
1	("Thymoma"[MeSH Terms] OR "Thymic epithelial tumor"[Supplementary Concept]) AND ("red cell aplasia, pure"[MeSH Terms] NOT "anemia, aplastic"[MeSH Terms])	Search by MeSH terms	154
2	(“thymoma”[Title/Abstract] OR “thymic carcinoma”[Title/Abstract] OR “thymic epithelial tumor”[Title/Abstract]) AND (“red cell aplasia”[Title/Abstract] NOT “aplastic anemia”[Title/Abstract])	Search by regular terms	284
3	#1 OR #2	Combination of the searches by union	318

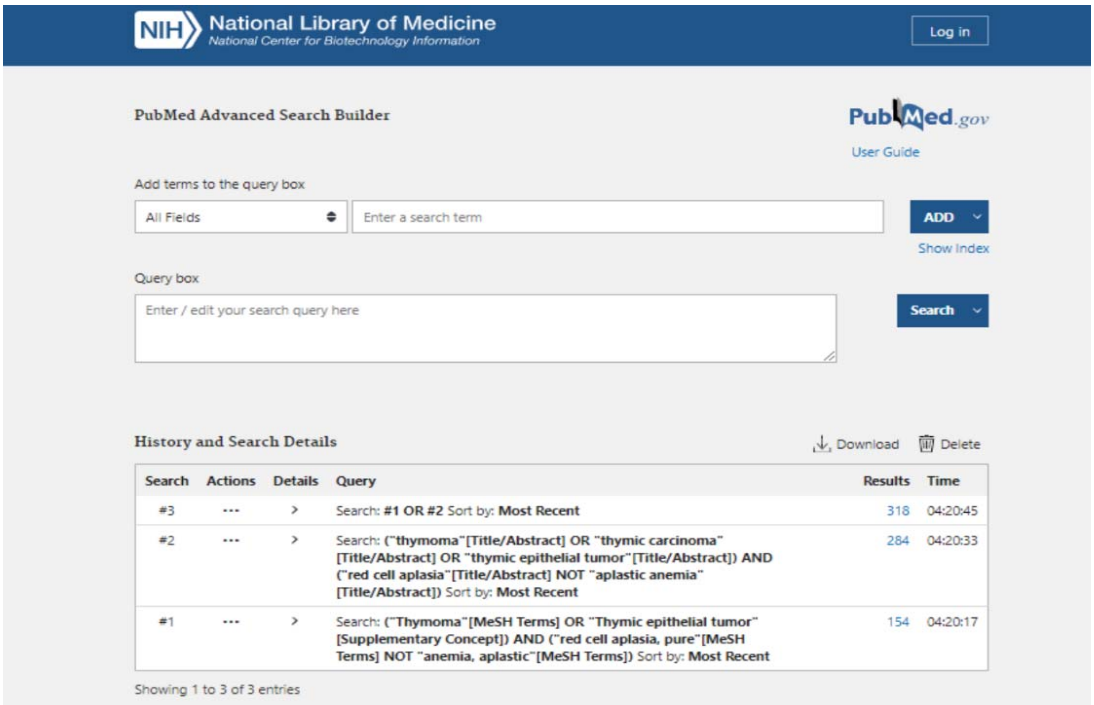

2. Embase®

https://www.embase.com/#search

Search time: 14/Feb/2021

Search number	Query	Note	Results
1	('thymoma'/exp OR 'thymic carcinoma'/exp)	Search of first keywords by Emtree terms	17,248
2	('pure red cell anemia'/exp NOT 'aplastic anemia'/exp)	Search of second keywords by Emtree terms	3,054
3	#1 AND #2	Combination of the searches by intersection	372
4	#3 AND [embase]/lim	Search results limited from Embase	313

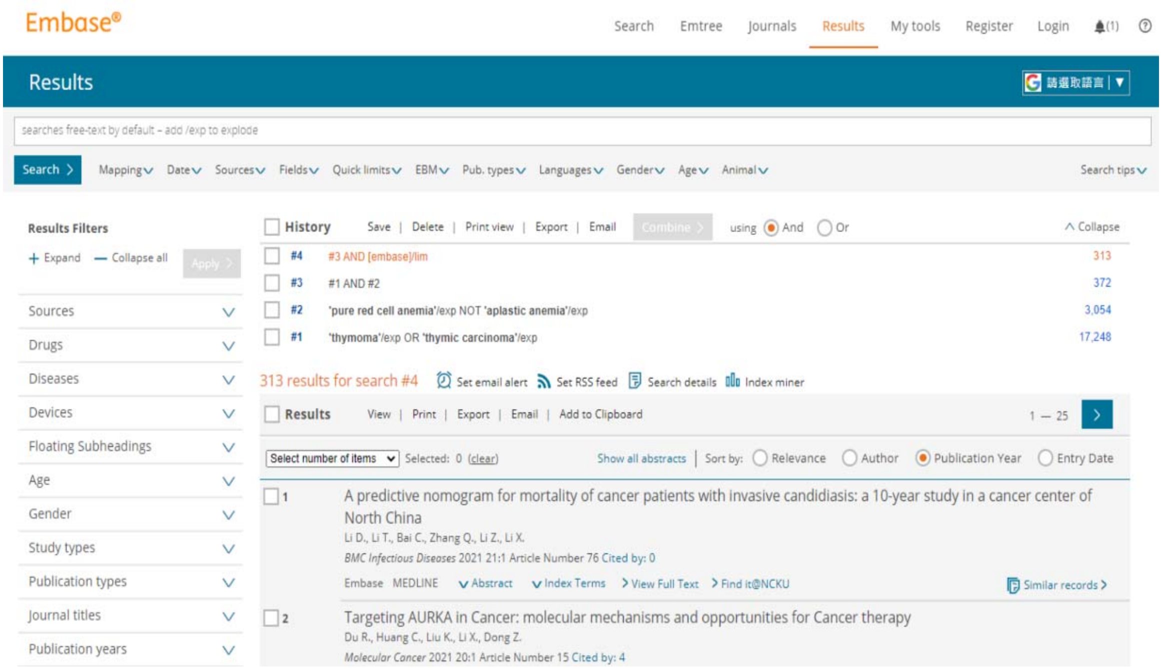

Supplementary Data 2: PRISMA checklist of the study

Section and Topic	Item #	Checklist item	Location where item is reported
**TITLE**			
Title	1	Identify the report as a systematic review.	p.1
**ABSTRACT**			
Abstract	2	See the PRISMA 2020 for Abstracts checklist.	p.3
**INTRODUCTION**			
Rationale	3	Describe the rationale for the review in the context of existing knowledge.	p.4
Objectives	4	Provide an explicit statement of the objective(s) or question(s) the review addresses.	p.4
**METHODS**			
Eligibility criteria	5	Specify the inclusion and exclusion criteria for the review and how studies were grouped for the syntheses.	p.5<br>Search strategies and systematic review of the literature
Information sources	6	Specify all databases, registers, websites, organisations, reference lists and other sources searched or consulted to identify studies. Specify the date when each source was last searched or consulted.	p.5<br>Search strategies and systematic review of the literature<br>and<br>Supp. Data 1
Search strategy	7	Present the full search strategies for all databases, registers and websites, including any filters and limits used.	p.5 and Supp. Data 1
Selection process	8	Specify the methods used to decide whether a study met the inclusion criteria of the review, including how many reviewers screened each record and each report retrieved, whether they worked independently, and if applicable, details of automation tools used in the process.	p.5<br>Search strategies and systematic review of the literature<br>and<br>Figure 1
Data collection process	9	Specify the methods used to collect data from reports, including how many reviewers collected data from each report, whether they worked independently, any processes for obtaining or confirming data from study investigators, and if applicable, details of automation tools used in the process.	p.5<br>Search strategies and systematic review of the literature

Section and Topic	Item #	Checklist item	Location where item is reported
			and<br>Figure 1
Data items	10a	List and define all outcomes for which data were sought. Specify whether all results that were compatible with each outcome domain in each study were sought (e.g. for all measures, time points, analyses), and if not, the methods used to decide which results to collect.	p.5<br>Search strategies and systematic review of the literature<br>and<br>Figure 1
	10b	List and define all other variables for which data were sought (e.g. participant and intervention characteristics, funding sources). Describe any assumptions made about any missing or unclear information.	p.5<br>Search strategies and systematic review of the literature<br>and<br>Figure 1
Study risk of bias assessment	11	Specify the methods used to assess risk of bias in the included studies, including details of the tool(s) used, how many reviewers assessed each study and whether they worked independently, and if applicable, details of automation tools used in the process.	p.5<br>Search strategies and systematic review of the literature<br>and<br>Figure 1
Effect measures	12	Specify for each outcome the effect measure(s) (e.g. risk ratio, mean difference) used in the synthesis or presentation of results.	p.5<br>Search strategies and systematic review of the literature
Synthesis methods	13a	Describe the processes used to decide which studies were eligible for each synthesis (e.g. tabulating the study intervention characteristics and comparing against the planned groups for each synthesis (item #5)).	p.5<br>Statistical analysis
	13b	Describe any methods required to prepare the data for presentation or synthesis, such as handling of missing summary statistics, or data conversions.	p.5<br>Statistical analysis
	13c	Describe any methods used to tabulate or visually display results of individual studies and syntheses.	Figure 1 and Supp. Data 2
	13d	Describe any methods used to synthesize results and provide a rationale for the choice(s). If meta-analysis was performed, describe the model(s), method(s) to identify the presence and extent of statistical heterogeneity, and software package(s) used.	NA; No meta-analysis was conducted

Section and Topic	Item #	Checklist item	Location where item is reported
	13e	Describe any methods used to explore possible causes of heterogeneity among study results (e.g. subgroup analysis, meta-regression).	NA; No meta-analysis was conducted
	13f	Describe any sensitivity analyses conducted to assess robustness of the synthesized results.	NA; No meta-analysis was conducted
Reporting bias assessment	14	Describe any methods used to assess risk of bias due to missing results in a synthesis (arising from reporting biases).	NA; No meta-analysis was conducted
Certainty assessment	15	Describe any methods used to assess certainty (or confidence) in the body of evidence for an outcome.	NA; No meta-analysis was conducted
**RESULTS**			
Study selection	16a	Describe the results of the search and selection process, from the number of records identified in the search to the number of studies included in the review, ideally using a flow diagram.	p.7 Systematic review of the literature and Figure 1
	16b	Cite studies that might appear to meet the inclusion criteria, but which were excluded, and explain why they were excluded.	Figure 1, Supp. Data 1 and Data 2
Study characteristics	17	Cite each included study and present its characteristics.	p.7 and Supp. Table 1
Risk of bias in studies	18	Present assessments of risk of bias for each included study.	p.7 and Supp. Table 1
Results of individual studies	19	For all outcomes, present, for each study: (a) summary statistics for each group (where appropriate) and (b) an effect estimate and its precision (e.g. confidence/credible interval), ideally using structured tables or plots.	Supp. Table 1
Results of syntheses	20a	For each synthesis, briefly summarise the characteristics and risk of bias among contributing studies.	p.7 Systematic review of the literature
	20b	Present results of all statistical syntheses conducted. If meta-analysis was done, present for each the summary estimate and its precision (e.g. confidence/credible interval) and measures of statistical heterogeneity. If comparing groups, describe the direction of the effect.	NA; No meta-analysis was conducted
	20c	Present results of all investigations of possible causes of heterogeneity among study results.	NA; No meta-analysis was conducted

frontiers

Section and Topic	Item #	Checklist item	Location where item is reported
	20d	Present results of all sensitivity analyses conducted to assess the robustness of the synthesized results.	NA; No meta-analysis was conducted
Reporting biases	21	Present assessments of risk of bias due to missing results (arising from reporting biases) for each synthesis assessed.	NA; No meta-analysis was conducted
Certainty of evidence	22	Present assessments of certainty (or confidence) in the body of evidence for each outcome assessed.	NA; No meta-analysis was conducted
**DISCUSSION**			
Discussion	23a	Provide a general interpretation of the results in the context of other evidence.	p.8-9
	23b	Discuss any limitations of the evidence included in the review.	p.9
	23c	Discuss any limitations of the review processes used.	p.9
	23d	Discuss implications of the results for practice, policy, and future research.	p.9
**OTHER INFORMATION**			
Registration and protocol	24a	Provide registration information for the review, including register name and registration number, or state that the review was not registered.	NA
	24b	Indicate where the review protocol can be accessed, or state that a protocol was not prepared.	NA
	24c	Describe and explain any amendments to information provided at registration or in the protocol.	NA
Support	25	Describe sources of financial or non-financial support for the review, and the role of the funders or sponsors in the review.	p.10
Competing interests	26	Declare any competing interests of review authors.	p.10
Availability of data, code and other materials	27	Report which of the following are publicly available and where they can be found: template data collection forms; data extracted from included studies; data used for all analyses; analytic code; any other materials used in the review.	p.10

From: Page MJ, McKenzie JE, Bossuyt PM, Boutron I, Hoffmann TC, Mulrow CD, et al. The PRISMA 2020 statement: an updated guideline for reporting systematic reviews. BMJ 2021;372:n71. doi: 10.1136/bmj.n71

Supplementary Figure 1: Swimmer plot for the case series

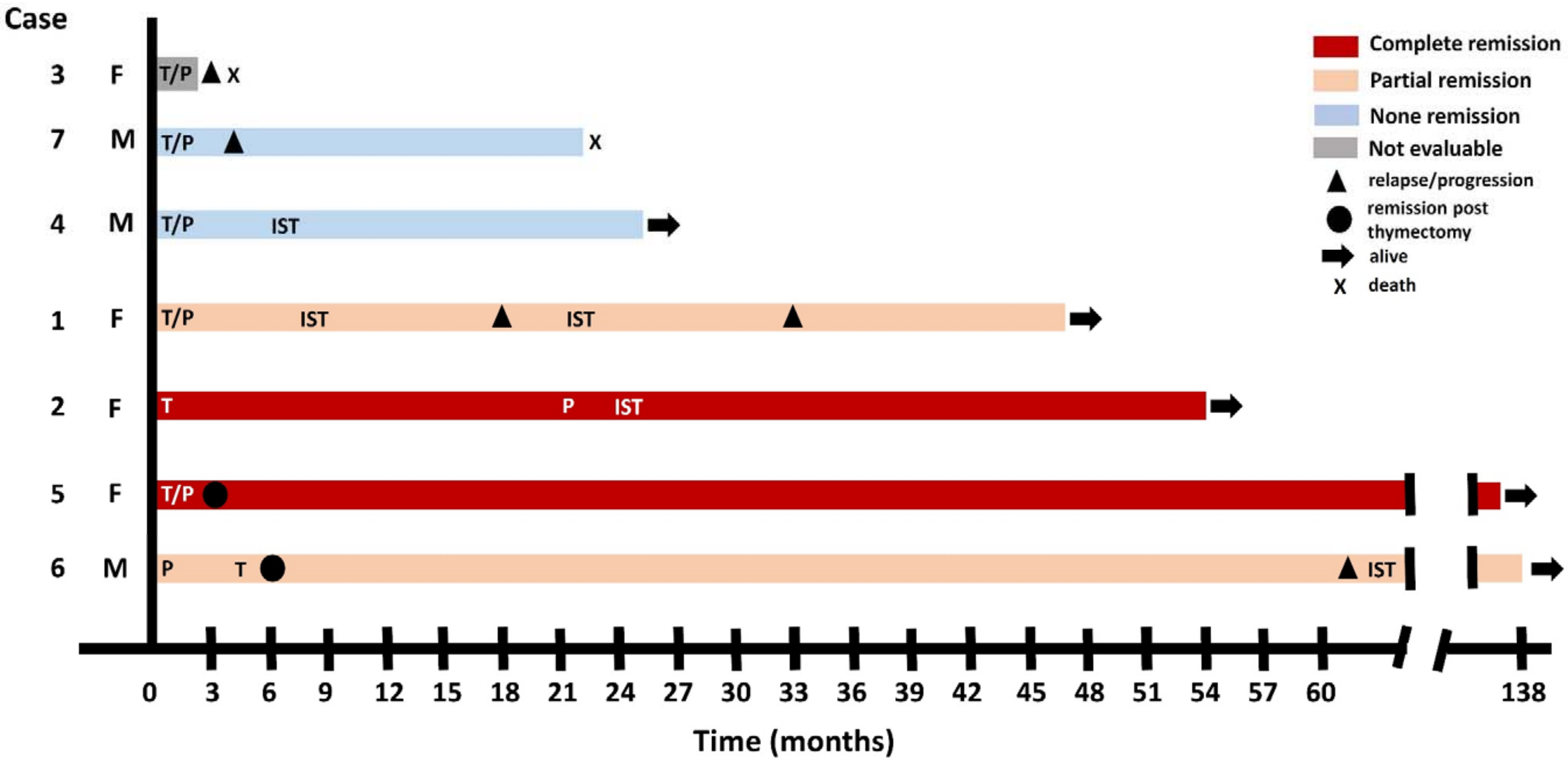

T/P, concurrent course of thymoma and pure red cell aplasia; T, onset of thymoma; P, onset of pure red cell aplasia; IST, immunosuppressive therapy; F, female; M, male

Supplementary Figure 2: Case demonstration

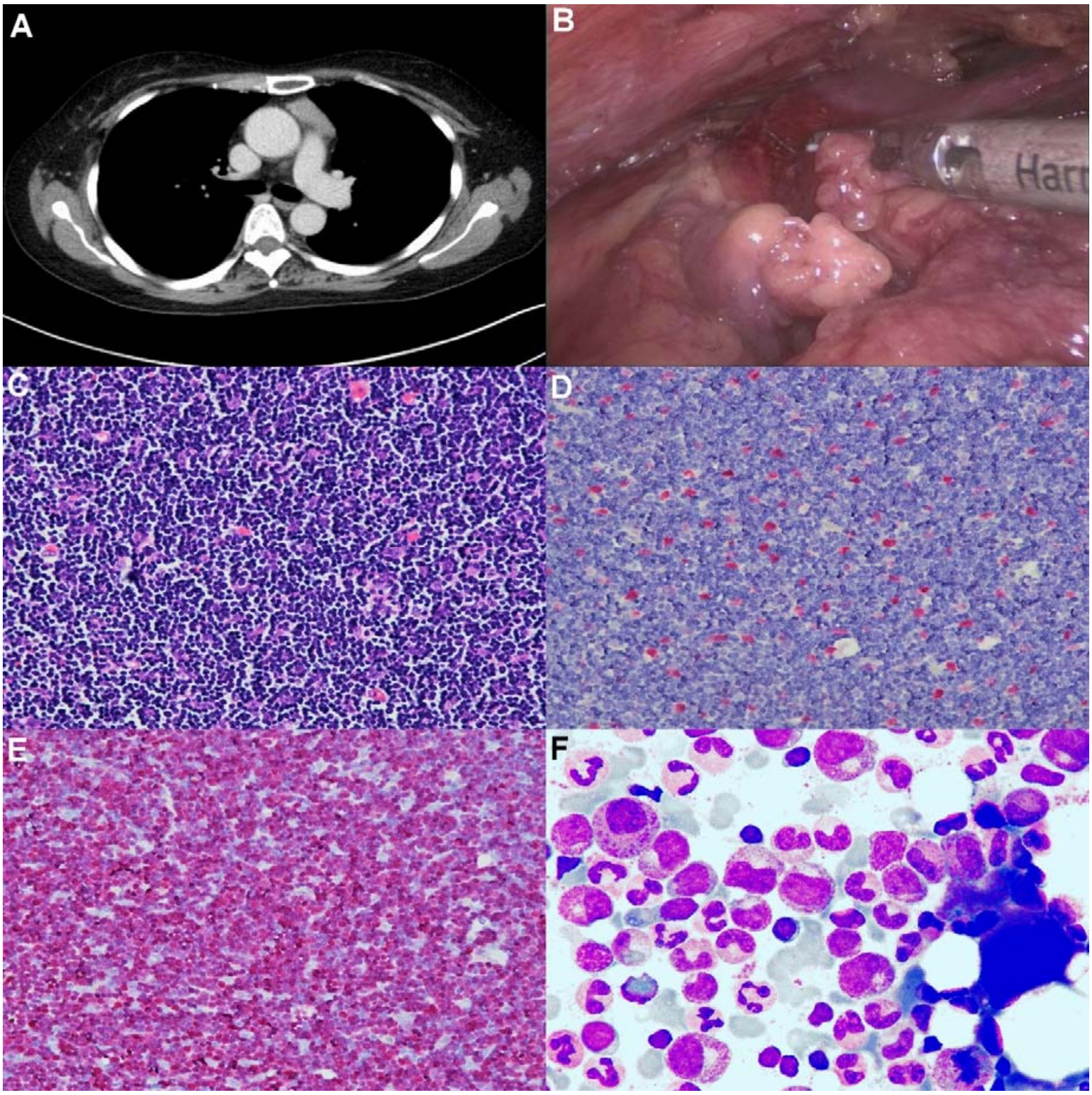

A 52-year-old female (Case 1) with thymoma-associated PRCA. (A) The chest CT image revealed an anterior solid and homogenous mediastinal tumor. (B) Video-assisted thoracosopic removal of the tumor. (C) Microscopic findings of the thymoma showed rare and unclustered epithelial cells surrounded by abundant lymphocytes, as highlighted by (D) p63-positive thymic epithelial cells interspersed with (E) TdT-positive lymphocytes. (F) Bone marrow smear showed marked erythroid hypoplasia (erythroid precursors less than 1% in the total nucleated cells), preserved granulocytic and megakaryocytic precursors, and occasional lymphocytic infiltrates, compatible to PRCA.

(C) Hematoxylin & Eosin, 400X; (D) p63, 400X; (E) TdT, 400X; (F) Liu's stain, 1000X

PRCA, pure red cell aplasia; CT, computed tomography; TdT, terminal deoxynucleotidyl transferase.

Supplementary Table 1: systematic review of the English language reports

Author	Year	Case number	Age and Sex	HDs	WHO Histology	Masaoka	Thymectomy	Remission
Suzuki	2005	4	51 F, 62 F, 69 F, 46 M	PRCA (n=4)	Thymoma (n=2); C (n=2)	NA	Yes (n=4)	Yes (n=3), no (n=1)
Yoshida	2005	1	78 F	PRCA	AB	NA	Yes	Yes
Fukushima	2006	1	33 M	PRCA	Thymoma	NA	Yes	Yes
Thompson	2006	13	median age of 65; M:F=7:6	PRCA (n=13)	AB (n=4), B1 (n=2), A (n=2), C (n=1); others (n=4)	NA	Yes (n=12)	Yes (n=4), no (n=8)
Lucchi	2007	1	43 M	PRCA and ITP	B1	IIB	Yes	Yes
Zaucha	2007	1	35 F	PRCA	AB	IVA	Yes	Yes
van der Marel	2007	1	56 M	PRCA and Good's syndrome	AB	NA	Yes	Yes (PRCA only); hypogammaglobulinemia persisted
Hirokawa	2008	41	Median age of 65; M (44%)	PRCA (n=41), AIHA (n=1)	A (n=1), B1 (n=3), B2 (n=4), AB (n=9); others (n=24)	NA	Yes (n=36)	Yes (≥PR, n=32), no (n=9)
Malhotra	2008	3	40 M, 61 M, 62 M	PRCA (n=3)	Thymoma	NA	Yes (n=1)	Yes (n=1), no (n=2)
Shiraishi	2008	1	55 F	PRCA, Good's syndrome	AB	I	Yes	No
Vohra	2008	2	58 M, 45 F	PRCA (n=2)	B1 (n=2)	I and IIA	Yes (n=2)	Yes (n=1), no (n=1)
Lin	2009	1	46 F	PRCA, Good's syndrome	AB	I	Yes	Yes (PRCA only); hypogammaglobulinemia persisted
Taniguchi	2009	1	57 F	PRCA, Good's syndrome	AB	II	Yes	Yes (PRCA only); hypogammaglobulinemia persisted
Kuribayashi	2010	1	79 F	PRCA, Good's syndrome	A	NA	Yes	Yes (PRCA only); hypogammaglobulinemia persisted
Jiang	2010	1/2[a]	60 F	PRCA	B3	NA	No (n=2)	No
Gadalla	2011	8/668[a]	Median age of 60; M (47%)	PRCA	NA	NA	NA	NA
Rosu	2011	1	80 F	PRCA	Thymoma	NA	Yes	Yes
Chen	2011	1	70 F	PRCA, Good's syndrome	AB	I	Yes	Yes (PRCA only); hypogammaglobulinemia persisted
Holbro	2012	2/29[a]	60 F, 67 M	PRCA (n=2)	A (n=1), AB (n=1)	NA	Yes	NA
Pena	2012	1	70 M	PRCA, Good's syndrome	Thymoma	NA	Yes	Yes (PRCA only); hypogammaglobulinemia persisted
Balikar	2013	1	55 F	PRCA	Thymoma	NA	Yes	Yes
Ck Rao	2013	1	38 F	PRCA	AB	I	Yes	No
Kojima	2013	1	64 F	PRCA	Thymoma	NA	Yes	Yes
Briones	2013	1	53 M	PRCA, Good's syndrome	B1	III	No	No
Fallahi	2014	1	54 F	PRCA	B1	NA	Yes	Yes
Gay	2014	1	31 M	PRCA, AAMT	Thymoma	III	Yes	Yes (bilineage)
Kobayashi	2014	1	81 F	PRCA	B1	IVA	Yes	Yes
Marmont	2014	1	55 F	PRCA	Thymoma	I	Yes	Yes
Rajan	2014	5/49[a]	Median age of 52; M (53%)	PRCA (n=5)	Thymoma (n=5)	IVA and IVB (n=5)	NA	NA
Hirokawa[b]	2015	41	Median age of 65; M (44%)	PRCA (n=41)	A (n=1), B1 (n=3), B2 (n=4), AB (n=9); others (n=24)	NA	Yes (n=36)	Yes (≥PR, n=27), no (n=14)
Fujiwara	2015	1	44 F	PRCA, AAMT	B1	IVA	Yes	Yes (bilineage)

Bernard	2016	2/85[a]	Median age of 58; M (46%)	PRCA (n=2)	Thymoma (n=2)	NA	NA	NA
Rivoisy	2016	11/36 [a]	Median age of 58; M:F=4:7	PRCA (n=11)	A (3%), B1-3 (47%), AB (25%), C (14%), NA (11%)[c]	I-II (50%)[c]	Yes (n=11)	Yes (≥PR, n=9), no (n=2)
Antar	2016	1	90 M	PRCA, Good's syndrome	B1	NA	No	Yes
Ambrogi	2016	1/13[a]	45 M	PRCA	B3	III	Yes	No
Onuki	2016	1	67 F	PRCA, AAMT	AB	III	Yes	Yes (bilineage)
Feinsilber	2017	1	34 F	PRCA	B1	IVA	Yes	No
Isshiki	2017	1	63 F	PRCA	B1	II	Yes	Yes
Li	2017	1	47 M	PRCA	AB	NA	Yes	Yes
Julka	2017	3/71[a]	Median age of 41; M (79%)	PRCA	Thymoma	NA	Yes (n=3)	NA
Okui	2017	1	50 M	PRCA and Good's syndrome	AB	I	Yes	Yes; hypogammaglobulinemia persisted
Moriyama	2018	8	Median age of 56; M:F=5:3	PRCA (n=7), PRCA and ITP (n=1)	A (n=1), B2 (n=5), B3 (n=2)	II (n=1), III (n=3), IVA (n=2), IVB (n=2)	Yes (n=8)	Yes (n=3), no (n=5)
Fu	2018	5/53[a]	Median age of 41; M (48%)	PRCA (n=5)	Thymoma (n=5)	NA	NA	Yes (≥PR, 81.1% in the total population)
Tabata	2018	1	40 F	PRCA	B2	IVA	No	No
Dahal	2018	1	60 M	PRCA and AAMT	B1	II	Yes	No
Cui	2019	1	60 M	PRCA	B3	NA	Yes	Yes
de Graaf	2019	1	80 M	PRCA	A	NA	Yes	No
Simkins	2019	1	61 F	PRCA, AAMT	B1	NA	Yes	Yes (trilineage)
Zaman	2019	8/78[a]	Median age 64; M:F=2:3	PRCA, Good's syndrome (n=8)	A (n=3), AB (n=2), C (n=3)	NA	Yes (n=8)	NA
Muratori	2020	1	60 M	PRCA progressed to AA	Thymoma	IV	Yes	No
Nicolas	2020	1/9[a]	44 M	PRCA and AA (n=1)	B2 (n=1)	I	Yes	Yes
Lo Iacono	2020	1	62 F	PRCA, AAMT	B3	NA	Yes	Yes (bilineage)
Chen	2020	3/12[a]	69 F, 76 M , 78 M	PRCA (n=3)	Thymoma (n=3)	NA	No (n=3)	Yes (n=3)
Lee	2020	1	79 F	PRCA	Thymoma	NA	No	No
Rubinstein	2020	1	57 F	PRCA	B2	IVA	Yes	No
Wang	2020	4	65 F, 53 F, 45 M, 38 M	PRCA, AIHA	AB (n=2), B2 (n=2)	I (n=2), IIA (n=1), III (n=1)	Yes	Yes (n=4)
Niparuck	2020	4/32[a]	median age of 57; M:F=1:3	PRCA (n=4)	B2 (n=2), B3 (n=2)	NA	Yes (n=1)	Yes (n=2), no (n=2)
Xavier	2020	1	58 M	PRCA	AB	I	Yes	Yes
Dragani	2020	1	62 M	PRCA, AAMT	B1	NA	Yes	No
Current study	2021	7	median age of 55; M:F=3:4	PRCA (n=4), PRCA and Good's syndrome (n=2), PRCA and MDS (n=1)	A (n=1), AB (n=4), B1 (n=1), B2 (n=1)	I (n=4), IIA (n=3)	Yes (n=7)	Yes (n=4), no (n=3); hypogammaglobulinemia persisted

a. Denotes the cases of thymoma-associated PRCA/total cases in the study.
b. A study derived from the identical cohort from Hirokawa, M. (2008).
c. Percentages presented by the total cases of thymoma in the study.
PRCA, pure red cell aplasia; HD, hematological disease; NA, not available; ITP, immune thrombocytopenia purpura; AIHA, autoimmune hemolytic anemia; CYC, cyclophosphamide; PR, partial remission; MDS, myelodysplastic syndrome; AAMT, acquired amegakaryocytic thrombocytopenia; AA, aplastic anemia.

References: (by chronological order)

Suzuki, S., et al., Myasthenia gravis accompanied by alopecia areata: clinical and immunogenetic aspects. Eur J Neurol, 2005. 12(7): p. 566-70.
Yoshida, S., et al., Effect of tacrolimus in a patient with pure red-cell aplasia. Clin Lab Haematol, 2005. 27(1): p. 67-9.
Fujishima, N., et al., Oligoclonal T cell expansion in blood but not in the thymus from a patient with thymoma-associated pure red cell aplasia. Haematologica, 2006. 91(12 Suppl): p. Ecr47.
Thompson, C.A. and D.P. Steensma, Pure red cell aplasia associated with thymoma: clinical insights from a 50-year single-institution experience. British Journal of Haematology, 2006. 135(3): p. 405-407.
Lucchi, M., et al., Four thymus-related syndromes in a case of invasive thymoma. The Journal of Thoracic and Cardiovascular Surgery, 2007. 134(5): p. 1376-1378.
van der Marel, J., et al., Thymoma with paraneoplastic syndromes, Good's syndrome, and pure red cell aplasia. J Thorac Oncol, 2007. 2(4): p. 325-6.
Zaucha, R., J.M. Zaucha, and J. Jassem, Resolution of thymoma-related pure red cell aplasia after octreotide treatment. Acta Oncol, 2007. 46(6): p. 864-5.
Hirokawa, M., et al., Long-term response and outcome following immunosuppressive therapy in thymoma-associated pure red cell aplasia: a nationwide cohort study in Japan by the PRCA collaborative study group. Haematologica, 2008. 93(1): p. 27-33.
Malhotra, P., et al., Spectrum of pure red cell aplasia in adult population of north-west India. Hematology, 2008. 13(2): p. 88-91.
Shiraishi, J., et al., Type AB thymoma accompanied by pure red cell aplasia and Good syndrome with CMV infection of tumor cells. Pathol Int, 2008. 58(8): p. 489-93.
Vohra, L.S., et al., Ectopic thymoma with pure red cell aplasia--ambiguity with indolence. Int J Surg, 2008. 6(6): p. e12-4.
Lin, C.S., et al., Pure red cell aplasia and hypogammaglobulinemia in a patient with thymoma. J Chin Med Assoc, 2009. 72(1): p. 34-8.
Taniguchi, T., et al., Good syndrome accompanied by pure red cell aplasia. Interact Cardiovasc Thorac Surg, 2009. 9(4): p. 750-2.
Jiang, D. and M. Wu, Thymectomy resulted in complete remission of pure red cell aplasia when associated with thymoma. Thorac Cardiovasc Surg, 2010. 58(4): p. 235-6.
Kuribayashi, K., et al., Pure red cell aplasia associated with Good's syndrome accompanied by decreased stem cell factor production in the bone marrow. Intern Med, 2010. 49(5): p. 377-82.
Gadalla, S.M., et al., A population-based assessment of mortality and morbidity patterns among patients with thymoma. Int J Cancer, 2011. 128(11): p. 2688-94.
Rosu, C., et al., Pure red cell aplasia and associated thymoma. Clinics and Practice, 2011. 1(1): p. 1.
Chen, L.-P., et al., Myelodysplasia followed by Good's Syndrome: A unique manifestation associated with thymoma. The Kaohsiung Journal of Medical Sciences, 2012. 28(4): p. 236-240.
Holbro, A., et al., High prevalence of infections and autoimmunity in patients with thymoma. Hum Immunol, 2012. 73(3): p. 287-90.
Pena, C., et al., [Association of Good syndrome with pure red cell aplasia: report of one case]. Rev Med Chil, 2012. 140(8): p. 1050-2.
Balikar, R., et al., Myasthenia gravis and pure red cell aplasia: a rare association. BMJ Case Rep, 2013. 2013.
Briones, J., et al., Thymoma associated with hypogammaglobulinaemia and pure red cell aplasia. Ecancermedicalscience, 2013. 7(364).
Ck Rao, A., B. Nayal, and C. Manohar, Pure red cell aplasia following thymothymectomy: a case report. Malays J Med Sci, 2013. 20(5): p. 83-5.
Kojima, M., et al., Deferasirox treatment improved hematopoiesis and led to complete remission in a patient with pure red cell aplasia. Int J Hematol, 2013. 98(6): p. 719-22.
Fallahi, S., M. Akbarian, and S. Dabiri, Pure red cell aplasia as a presenting feature in systemic lupus erythematosus and association with thymoma, hypothyroidism and hypoparathyroidism: a case report and literature review. Iran J Allergy Asthma Immunol, 2014. 13(2): p. 138-43.
Gay, C.M., et al., Thymoma complicated by acquired amegakaryocytic thrombocytopenia and pure red cell aplasia. J Natl Compr Canc Netw, 2014. 12(11): p. 1505-9.
Kobayashi, R., et al., Recurrent thymoma with stiff-person syndrome and pure red blood cell aplasia. Ann Thorac Surg, 2014. 97(5): p. 1802-4.
Marmont, A.M., et al., Systemic lupus erythematosus complicated with thymoma and pure red cell aplasia (PCRA). CR of both complications following thymectomy and allogeneic haematopoietic SCT (HSCT), but persistence of antinuclear antibodies (ANA). Bone Marrow Transplant, 2014. 49(7): p. 982-3.
Rajan, A., et al., Cixutumumab for patients with recurrent or refractory advanced thymic epithelial tumours: a multicentre, open-label, phase 2 trial. The Lancet Oncology, 2014. 15(2): p. 191-200.
Fujiwara, A., et al., Myasthenic crisis caused by preoperative chemotherapy with steroid for advanced thymoma. Ann Thorac Surg, 2015. 99(1): p. e11-3.
Hirokawa, M., et al., Long-term outcome of patients with acquired chronic pure red cell aplasia (PRCA) following immunosuppressive therapy: a final report of the nationwide cohort study in 2004/2006 by the Japan PRCA collaborative study group. Br J Haematol, 2015. 169(6): p. 879-86.
Ambrogi, M.C., et al., Pleural recurrence of thymoma: surgical resection followed by hyperthermic intrathoracic perfusion chemotherapy†. Eur J Cardiothorac Surg, 2016. 49(1): p. 321-6.
Antar, A.I., et al., Thymoma with Concomitant Pure Red Cell Aplasia, Good's Syndrome and Myasthenia Gravis Responding to Rituximab. Indian J Hematol Blood Transfus, 2016. 32(Suppl 1): p. 219-22.
Bernard, C., et al., Thymoma associated with autoimmune diseases: 85 cases and literature review. Autoimmunity Reviews, 2016. 15(1): p. 82-92.
Onuki, T., et al., Invasive thymoma with pure red cell aplasia and amegakaryocytic thrombocytopenia. Hematology Reports, 2016. 8(4).

Rivoisy, C., et al., Thymic Epithelial Tumor-Associated Cytopenia: A 10-Year Observational Study in France. J Thorac Oncol, 2016. 11(3): p. 391-9.
Feinsilber, D., K.A. Mears, and B.L. Pettiford, Polyparaneoplastic Manifestations of Malignant Thymoma: A Unique Case of Myasthenia, Autoimmune Hepatitis, Pure Red Cell Aplasia, and Keratoconjunctivitis Sicca. Cureus, 2017. 9(6): p. e1374.
Isshiki, Y., et al., Cyclosporine is a potential curative treatment option for advanced thymoma. Exp Hematol Oncol, 2017. 6: p. 13.
Julka, P.K., et al., Outcomes of thymoma treated with multimodality approach: a tertiary cancer center experience of 71 patients. Tumori, 2017. 103(6): p. 572-576.
LI, P., et al., Cyclosporin A Reversed Chemoresistance of a Patient with Pure Red Cell Aplasia Secondary to Thymoma. 2017. 37(8): p. 4611-4614.
Okui, M., et al., Pure Red Cell Aplasia Associated with Good Syndrome. Korean J Thorac Cardiovasc Surg, 2017. 50(2): p. 119-122.
Dahal, S., et al., Acquired Amegakaryocytic Thrombocytopenia and Pure Red Cell Aplasia in Thymoma. Case Rep Hematol, 2018. 2018: p. 5034741.
Fu, R., et al., The clinical characteristics and therapy response of patients with acquired pure red cell aplasia. Hematology, 2018. 23(9): p. 639-645.
Moriyama, S., et al., Pure red cell aplasia associated with thymoma: a report of a single-center experience. 2018, 2018. 10(8): p. 5066-5072.
Tabata, R., et al., Progressive peripheral CD8+ T lymphocytosis complicated by pure red cell aplasia following immunosuppressive therapy for thymoma-associated myasthenia gravis. International Immunopharmacology, 2018. 63: p. 14-18.
Cui, X., et al., Occurrence of acute pulmonary embolism induced by recombinant erythropoietin during treatment of pure red cell aplasia associated with thymoma: A case report. Medicine (Baltimore), 2019. 98(10): p. e14789.
de Graaf, M.A., et al., A ring-calcified thymoma, mimicking pericardial cyst, precedes pure red cell aplasia for more than 10 years - A case-based overview of pathophysiology. Neth J Med, 2019. 77(10): p. 373-376.
Simkins, A., et al., Acquired amegakaryocytic thrombocytopenia and red cell aplasia in a patient with thymoma progressing to aplastic anemia successfully treated with allogenic stem cell transplantation. Hematol Oncol Stem Cell Ther, 2019. 12(2): p. 115-118.
Zaman, M., et al., Clinical and laboratory features of seventy-eight UK patients with Good's syndrome (thymoma and hypogammaglobulinaemia). Clin Exp Immunol, 2019. 195(1): p. 132-138.
Chen, Z., et al., Immunosuppression therapy is effective for both acquired tumor-associated and primary pure red cell aplasia: a match pair case-control study. Ann Hematol, 2020. 99(7): p. 1485-1491.
Dragani, M., et al., Pure red cell aplasia and amegakaryocytic thrombocytopenia in thymoma: The uncharted territory. Clin Case Rep, 2020. 8(4): p. 598-601.
Lee, S.Y., A. Gill, and S.M. Jung, Pure red cell aplasia and seronegative myasthenia gravis in association with thymoma. J Community Hosp Intern Med Perspect, 2020. 10(3): p. 238-241.
Lo Iacono, G., et al., Thymoma and pure red cell aplasia with hypoplasia of megakaryocytopoiesis: A rare and life-treating condition. Transfus Apher Sci, 2020. 59(2): p. 102656.
Muratori, L., et al., Discrepancy between tumor response and hematologic response in a patient with thymoma and aplastic anemia treated with ciclosporin. Mediastinum, 2020. 4.
Nicolas, G., et al., Aplastic anemia related to thymoma: a survey on behalf of the French reference center of aplastic anemia and a review of the literature. Haematologica, 2020. 105(7): p. e333-e336.
Niparuck, P., et al., Etiologies and Treatment Burden in Adult Patients with Pure Red Cell Aplasia: A Single-Center Experience and Review of Literature. Anemia, 2020. 2020: p. 4812759.
Rubinstein, M.M., et al., Management of thymoma-associated pure red cell aplasia: A novel use of blood substitute HBOC-201 in a Jehovah's Witness. Clin Case Rep, 2020. 8(2): p. 289-292.
Wang, W., et al., Coexistence of Pure Red Cell Aplasia and Autoimmune Haemolytic Anaemia Associated with Thymoma. Acta Haematologica, 2020. 143(5): p. 491-495.
Xavier, R.D., et al., Thymoma associated with pure red cell aplasia: a case report and literature review. Indian J Thorac Cardiovasc Surg, 2020. 36(4): p. 404-408.
